# Adaptation and pre-test of a shortened Stepping Stones and Creating Futures intervention focused on HIV for young men in rural South Africa

**DOI:** 10.1371/journal.pgph.0001632

**Published:** 2023-02-24

**Authors:** Andrew Gibbs, Dumsani Gumede, Oluwafemi Adeagbo, Yandisa Sikweyiya, Esnat Chirwa, Smanga Mkhwanazi, Manono Luthuli, Zakhele Xulu, Carina Herbst, Thembelihle Zuma, Siphesihle Hlongwane, Nonhlanhla Okesola, Jaco Dreyer, Sivuyile Khaula, Laura Washington, Maryam Shahmanesh

**Affiliations:** 1 Department of Psychology, University of Exeter, Exeter, United Kingdom; 2 Gender and Health Research Unit, South African Medical Research Council, Pretoria, South Africa; 3 School of Nursing and Public Health, University of KwaZulu-Natal, Durban, South Africa; 4 Institute for Global Health, University College London, London, United Kingdom; 5 Africa Health Research Institute, Somkhele, KwaZulu-Natal, South Africa; 6 Department of Community and Behavioral Health, College of Public Health, University of Iowa, Iowa, United States of America; 7 Department of Sociology, University of Johannesburg, Johannesburg, South Africa; 8 School of Public Health, University of the Witwatersrand, Johannesburg, South Africa; 9 Project Empower, Durban, South Africa; Zuckerberg San Francisco General Hospital and Trauma Center, UNITED STATES

## Abstract

Men’s engagement in HIV prevention and treatment is suboptimal, including in South Africa. We sought to address this through adapting an evidence-based intervention, Stepping Stones and Creating Futures (SSCF), to strengthen its HIV content and provide a more scalable (shorter) intervention in rural South Africa. We then conducted a mixed methods pre-test of the intervention among young men aged 18–35 years. To adapt SSCF, we reviewed the current evidence base and worked with male Peer Navigators to update the SSCF theory of change (ToC) and manual. The revised intervention was ~45 hours (9 sessions) as opposed to ~63 hours and included a greater focus on HIV prevention and treatment technologies. Overall, 64% (*n* = 60) of men approached agreed to participate in the intervention, uptake (attending one session) among those who agreed was n = 35(58%) and retention (attending 6 or more sessions) was n = 25(71%). Qualitative data emphasized the intervention was acceptable, with young men describing it as something they liked. The qualitative data also broadly supported the intervention ToC, including the normalization of HIV in men’s lives, and the importance of health for men in achieving their life goals. However, it also highlighted the need to focus more on HIV-related stigma and fear, and the importance of HIV self-testing kits in encouraging testing. We revised the ToC and manual in light of this data. The adapted SSCF is acceptable and supports the ToC. Next steps is an evaluation to look at effectiveness of the intervention.

## Introduction

Despite rapidly expanding HIV treatment globally, and advances in biomedical HIV prevention technologies, young men continue to have poor engagement in the HIV treatment and prevention cascades [1]. This inadequate engagement leads to high HIV related mortality amongst men and high HIV incidence amongst adolescent girls and young women. In 2017, the UNAIDS Blind Spot report [1] emphasized the urgent need to engage with, and improve, men’s HIV testing and linkage to prevention or treatment services.

The pattern of young men’s limited engagement in HIV treatment and prevention is also seen in South Africa where, despite free universal testing and treatment since 2016, men continue to have poor HIV outcomes [2, 3]. Nationally, young men (aged 25–34 years) have the lowest viral suppression (41.5%) of any group [2]. Additionally, use of HIV prevention technologies is low: only 38% of South African men report condom use at last sex, and in KwaZulu-Natal only 38% of men aged 15 and older report voluntary medical male circumcision (VMMC), compared to 62% nationally [2].

One reason for men’s inadequate engagement in the HIV prevention and treatment cascades is the structure of health systems. Healthcare systems often prioritize mother and child health, passively excluding men from care [4] and HIV testing and treatment is primarily targeted at women in antenatal settings [4]. Furthermore, studies suggest men may feel healthcare spaces are ‘women’s spaces’ limiting men’s willingness to seek HIV care [5, 6]. Finally, primary care, including HIV testing and treatment, is often hard to access for everyone with long queues and inconvenient opening hours [6] as well as concerns about treatment by healthcare staff, including whether patients’ privacy will be maintained [7].

Another reason for men’s low engagement in HIV testing, prevention and treatment are men’s gender inequitable norms of masculinity. Gender inequitable norms of masculinity broadly refers to a set of practices and attitudes linked to male dominance and control over women [8], which can include heavy alcohol use, seeking multiple sexual partners, and the use of violence against women [9], as well as often antagonistic and violent relationships with other men [10]. Such norms emerge from contexts of poverty, men’s own experiences of violence and adverse events, particularly in childhood, as well as dominant notions and expectations around masculinity, rooted in patriarchy and local histories of violence and colonialism [8, 11]. Such inequitable norms of masculinity reduce men’s access to HIV treatment and prevention technologies through a range pathways, including men’s unwillingness to acknowledge their vulnerability limiting healthcare seeking [12], as well as the fear of an HIV-positive diagnosis challenging their idealized masculinity [13, 14].

Several interventions have sought to address men’s gender inequitable masculinities as a pathway to improve HIV prevention and treatment outcomes [15–17]. Many of these interventions are based on small-group participatory approaches, drawing on Freire’s [18] understanding of adult education. Studies have shown varying degrees of success using this approach [19].

A common intervention focused on addressing gender inequitable norms to support HIV prevention and treatment is the Stepping Stones intervention. Developed in the 1990s in Uganda [20], it has been used widely with women and men, including in the delivery of the DREAMS programming across 15 countries sub-Saharan Africa [21]. Evaluations have shown a range of positive outcomes [22].

Recently Stepping Stones was combined with a livelihoods intervention called Creating Futures (together Stepping Stones and Creating Futures—SSCF) [23] to simultaneously address poverty and inequitable gender norms. The combined intervention was evaluated among young (ages 18–30 years) women and men in urban informal settlements in South Africa through a cluster randomized controlled trial (RCT). After two-years SSCF showed a positive impact on women’s and men’s livelihoods, and for men a reduction in their perpetration of intimate partner violence (IPV) and alcohol use–in this evaluation women and men were not in relationships [23]. In qualitative research, men described a range of pathways through which SSCF worked, including reducing shame, and building relationships with other men, allowing them to reflect on and modify their masculinities towards more health enhancing and gender equitable masculinities [24, 25].

While SSCF addressed men’s gender inequitable masculinities, there remained challenges with it as an intervention model for HIV related outcomes. First, SSCF showed no impact on HIV prevention and treatment outcomes: there was no increase in HIV-testing or improvements in treatment adherence, and qualitative data showed no change in use of biomedical HIV prevention technologies. Second, SSCF is long, comprising of 21 sessions (63 hours contact time), with two sessions delivered every week. The length of the intervention was shaped by adult education theory that emphasizes the need for time to reflect and integrate new learnings into daily life [26]. However, in the RCT of SSCF retention across the intervention was low, with work seeking and mobility impacting this [27]. The length further impacts on the ability for the intervention to be delivered at scale.

To overcome the challenge of young men’s poor engagement in the HIV treatment and prevention cascades, we sought to co-adapt SSCF with young men in rural KwaZulu-Natal, South Africa. Co-adaptation refers to working very closely with those ‘targeted’ by an intervention to adapt an existing intervention. We also sought to assess whether the adapted intervention was suitable for formal evaluation.

To provide a structure to the project we applied the UK Medical Research Council’s “Developing and Evaluating Complex Interventions” [28, 29] framework, which advocates using realist evaluation to: (1) clarify the causal assumptions underpinning the theory of change (2) refine the theoretical basis of the intervention (3) describe the intervention in full and develop a manual; and (4) understand uptake and retention of the intervention, and (5) refine outcome measurement. In the discussion we then reflect on whether or not the adapted intervention adequately achieved the five aspects of the MRC framework and is thus suitable for more formal evaluation.

## Methods and materials

This was a joint project between the African Health Research Institute (AHRI), the South African Medical Research Council (SAMRC), University College London, and Project Empower, a non-government organisation (NGO) based in KwaZulu-Natal, South Africa. Project Empower (https://projectempower.org.za) have implemented the SSCF intervention widely, including in the aforementioned trial, and have extensive experience of intervention adaptation and working with young men in South Africa.

We operationalized the MRC’s Complex Interventions framework through three steps: 1) co-adaption of the SSCF theory of change (ToC) and intervention manual; 2) a mixed-methods pre-test of the intervention; 3) a final revision of the ToC and intervention manual.

### Context

The study was conducted in the Hlabisa sub-district of uMkhanyakude district in KwaZulu-Natal, South Africa. This is the site of the AHRI Health Demographic Surveillance System (HDSS) [30] which provides an annual census of people residing in the area. The area is primarily rural, though also has a number of smaller communities with denser housing, and one large town, which young people often migrate to. Overall, only 18% of those aged 18–35 years who are not in school are in full-time employment, and two-thirds of households receive social grants. There are also high-levels of circular-migration to larger urban centres and back again. Among men aged 15 and above HIV-prevalence is 25% [31].

Co-developers were young male Peer Navigators from the area and aged 18–30 years, employed by AHRI. The Peer Navigators had helped develop and implement the Thetha Nami intervention [32], which is an intervention seeking to address the wider sexual and reproductive health challenges young people faced [32]. Thetha Nami comprises of two components, the first is provision of youth friendly healthcare services through mobile clinics and fixed clinics. The second component are the Peer Navigators, who work with other young people to support them to access services, as well as distribute basic healthcare products to them, typically through one-on-one support and engagement. Peer Navigators have completed high school and been selected by local and traditional authorities in the area. They have been trained in HIV and sexual health promotion. They receive ongoing supervision and oversight by a team which includes professional nurses, social scientists and counsellor [32]. As such, Peer Navigators had a good sense of the challenges other young men faced in relation to health systems and wider social and economic issues. In total 10 male Peer Navigators were involved in this project.

#### i) Co-adaptation of SSCF Theory of Change and intervention manual

The co-adaption of the ToC and manual was done in three stages. First, the research team developed an initial ToC drawing on their previous research and broader published literature. Second, we worked with male Peer Navigators who were part of Thetha Nami to co-develop and refine the ToC (week 1) and orientate them to the intervention approach (week 2). Third, we refined the ToC based on their feedback, adapted sessions or developed new sessions and finalized a manual for pre-testing.

#### ii) Pre-test of the intervention

We conducted a mixed methods ‘pre-test’ of the adapted SSCF manual within the context of a youth friendly mobile clinic health systems of the Thetha Nami intervention. Our focus of the pre-test was on collecting adequate data to reflect on the MRC’s five aspects of complex interventions.

*Participants*. For the pre-test we recruited men aged 18–35 years who lived within the AHRI HDSS. Our sample were men living in ‘week-blocks’ near to where the intervention would be delivered. Week-blocks refer to an area the AHRI fieldwork team can cover in a week. Inclusion criteria included: i) being aged 18–35 years old, ii) normally resident in the selected week-block, defined as spending four or more nights a week in the area, iii) able to speak English or isiZulu, iv) willing to provide informed consent.

Participants were recruited in two ways. First, we used the HDSS as a sampling frame to randomly select 100 men matching the inclusion criteria and living within the selected ‘week-blocks’. Trackers (fieldworkers who identify community residents) were provided with a list of selected men and went to homes to identify and confirm information and invite them to participate in the study/intervention. If they agreed they provided informed consent.

Second, the Peer Navigators from Thetha Nami used their peer networks to recruit men meeting the inclusion criteria, from within the same community areas. Peer recruitment was done two days prior to intervention delivery, and on the first two days of intervention delivery. Men who were interested in participating, came to where the intervention was being delivered and prior to commencing, provided informed consent to participate.

*Intervention*. The intervention delivered in the pre-test was the co-adapted manualised version of SSCF developed in step 1. The intervention was 9 sessions, comprising approximately 45 hours contact time. The intervention was designed to be delivered to between 10 and 20 young men at a time, using small group participatory activities. SSCF was delivered in the context of being able to access youth friendly healthcare through the ongoing Thetha Nami intervention (described above). All intervention participants were issued with slips providing information on how to access mobile clinics (e.g., through sending a message requesting a call-back), and slips with QR codes linked participants to their data, although individuals could access without these. Participants were also shown the mobile clinics and a nurse explained the process and care provided.

*Intervention delivery*. The pre-test was designed to be done twice, with different groups of men. First, in a ‘short-delivery’ format, SSCF was implemented over 9 days in a two-week period (essentially every working day). Second, after a pause to modify the manual based on initial experience and feedback, SSCF was delivered in a ‘long-delivery’ format to a new group of men. Long-delivery of SSCF was over a 4-week period (normally two sessions per week).

*Implementation*. The intervention was implemented by a trained male Project Empower facilitator, of similar age and background to the men recruited. He had previously delivered SSCF in various projects and led training of trainer processes for the intervention and had run the co-adaptation process with the Peer Navigators. Two Peer Navigators were in attendance during both interventions.

### Data collected

**Fig 1:**
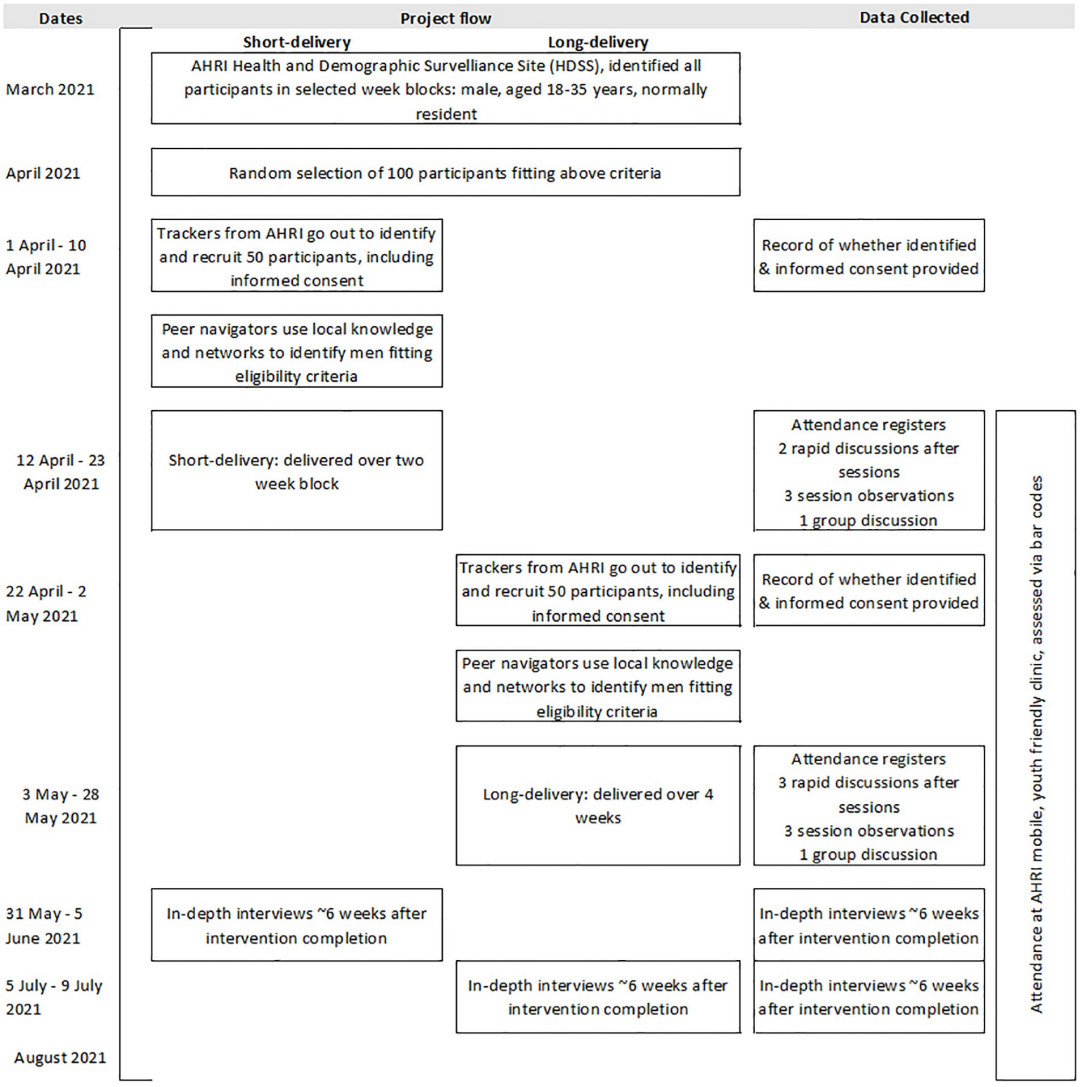
Timeline of the ‘pre-test’ of short- and long-delivery format Stepping Stones and Creating Futures intervention, with participant data collected.

During the pre-test quantitative and qualitative data (Fig 1) were collected to enable assessment against the MRC’s five aspects of evaluating and developing complex interventions framework.

#### Qualitative data

A range of qualitative data were collected throughout the pre-test (see Fig 1). In short, in both formats we observed three sessions, we conducted rapid discussions with participants after sessions (n = 2 short-delivery; n = 3 long-delivery), and in both formats we conducted one group-discussion at the completion of all sessions. Additionally, six weeks after the completion of the intervention, six participants per group, selected for an age mix, participated in in-depth interviews.

Topic guides for rapid discussions and group discussions focused on immediate feedback about key learnings for participants, what they enjoyed about the session and what they did not. In-depth interviews after six weeks focused more widely on challenges and experiences of attendance and whether or not they had accessed clinics or healthcare.

Qualitative analysis included transcription and translation into English, with quality checking. We conducted thematic network analysis [33], focused on understanding the extent to which men’s experiences of the intervention fit into the theory of change used to develop the intervention, and potential challenges and limitations of this.

#### Quantitative data

To understand the possibilities of recruitment and to assess uptake and retention in the intervention, we calculated a range of different quantitative measures. We first calculated the percentage who agreed to participate in the study, defined as the number who provided informed consent divided by number identified by trackers. Uptake of intervention, as a percentage, was calculated as the number of participants who attended at least one session divided by number who provided informed consent for the study. Finally, intervention retention was defined as the number who attended six or more sessions divided by number who attended at least one session.

We assessed uptake of the intervention and retention in the intervention by recruitment strategy (random versus peer network) as well as intervention format (short-delivery versus long-delivery). To estimate difference between groups, we used a Fishers Exact Test, or Pearson’s Chi-Squared Test, depending on cell sizes.

*Outcome measurement*. we assessed whether an outcome measurement of linkage to a clinic, via QR-coded slip, was possible for future studies. We assessed this through ongoing data collection at clinics and through qualitative data to understand if other relevant outcome measurements were missed.

#### iii) Final revision of the ToC and intervention manual of SSCF

The third step was to revise the SSCF ToC and implementation manuals based on data collected during the pre-test. We reviewed all the data and revised the ToC, manual and causal assumptions based on this.

*COVID-19*. The project was implemented during the COVID-19 pandemic and this had several impacts on the planned process. Specific changes included that the intervention was delivered by a Project Empower facilitator, rather than Peer Navigators, who we had hoped would be trained and supported to deliver the intervention. We also delivered the pre-test in AHRI’s main site, rather than a community-based facility. This allowed greater control around COVID-19 protocols but may have limited people’s openness to attend. The face-to-face work was conducted between COVID-19 peaks, and face-masks and hand-sanitiser were provided and the room was well ventilated, and daily symptom screening was conducted.

*Ethics*. The study received ethical approval from the University College London’s ethics committee (5672/004), the Biomedical Research Ethics Committee at the University of KwaZulu-Natal (RECIP010/2020), and the South African Medical Research Council’s ethics committee (EC010-5/2020). It was also approved by AHRI’s community oversight committee. All participants provided written informed consent to participate in the study.

## Results

We discuss the results of the project in the three steps, first the process of adapting and co-developing the ToC, second the pre-test and finally revisions based on the pre-test.

### Step 1: Co-adaption of the SSCF theory of change (ToC) and intervention manual

**Fig 2:**
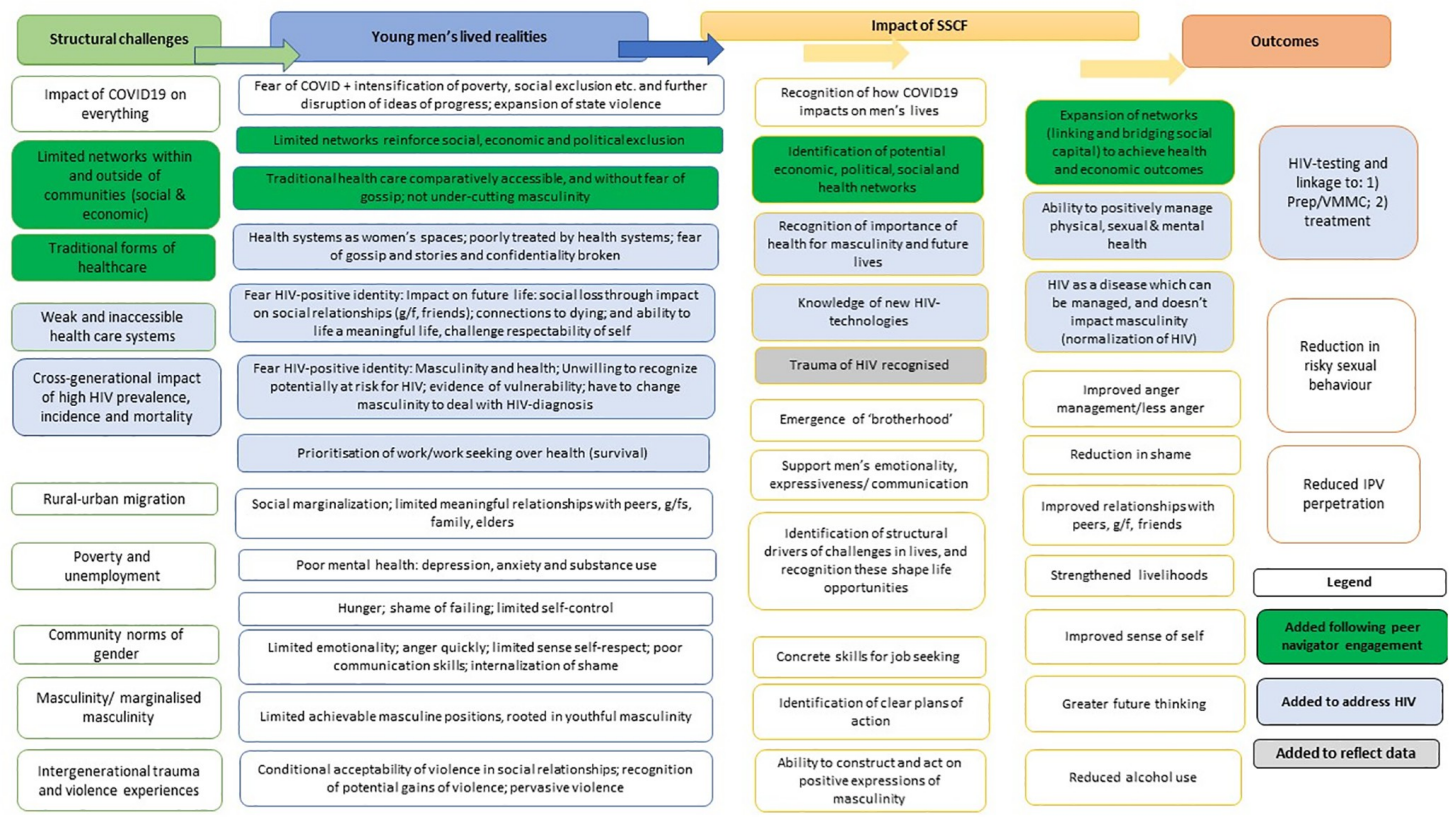
Theory of Change for Stepping Stones and Creating Futures including its changes over the project process.

We initially developed the ToC (Fig 2) based on our prior qualitative and quantitative research on SSCF [25, 34, 35], including discussion with Project Empower implementers, and reviews of evidence of effective violence prevention interventions [36]. The ToC sought to describe how the structural challenges young men faced shaped young men’s lived realities, and how SSCF impacted on these to reduce men’s perpetration of IPV, alcohol use and strengthen livelihoods (clear boxes in Fig 2).

To strengthen the response to HIV, we integrated learnings from AHRI’s own work [13, 32, 37], on men, masculinities, and HIV (blue boxes in Fig 2), as well as the wider evidence-base [19]. Issues included the impact of HIV on masculinity, the challenge of accessible primary healthcare services and HIV-related stigma.

We then worked with the Peer Navigators to clarify and extend the ToC. These clarifications were important because of the rural context of the project area, compared to urban informal settlements where SSCF data came from. To do this we used a ‘Problem Tree’ analysis. In a Problem Tree, the roots refer to the underlying drivers/causes, the trunk the problem, and the leaves/branches are the impacts of the problem. There was also a focus on what Peer Navigators could contribute to solving the challenges they identified and what their experiences of running Thetha Nami could contribute.

Peer Navigators reinforced that unemployment was a major challenge, and that young men felt shame and pressure if they could not work and support themselves and their families. They also described how alcohol and violence were common experiences among men. There was also much discussion about the impact of HIV in their communities and the challenges young men faced in accessing HIV testing, treatment and prevention technologies.

The Peer Navigators then focused on what they felt could be done in an intervention to address these challenges and HIV. After much debate, they highlighted five areas:

1. Stigma of testing for HIV
2. The role of religion and culture in the fight against HIV
3. Problems in relationships
4. Role played by parents in children’s lives
5. Tools used to assist youth to be free from HIV (PrEP, PEP, ART, and HIV self-testing kits)

The Peer Navigators raised two issues that were not in the initial ToC. The first was their use of traditional healthcare systems as an alternative to biomedical healthcare. Traditional healthcare was discussed in terms of being more accessible than biomedical healthcare, less likely to lead to gossip being spread, as well as being something ‘men’ have been practicing for a long time. The second issue they raised was the lack of networks within the community and outside of it, and how this impacted on their economic opportunities. These were integrated into the ToC (green boxes in Fig 2).

In the second week of the co-development workshop we provided the Peer Navigators with an outline of SSCF and and focused on how sections could be adapted and developed. An important emphasis was on clarifying learning approaches as dialogical (discussion-based) rather than didactic (lectures) and providing an overview of sessions. Several sessions from SSCF were also run with participants, picking up on key areas raised the previous week, and there was discussion about what may change in sessions to make it relevant to the area they lived and worked in. A key issue raised was a greater focus on role plays and dramas as an approach to learning.

Based on the revised ToC we identified potential new impacts of SSCF (in darker grey) on the ToC, Fig 2) that sought to address the additional aspects of HIV in young men’s lives. This included stand-alone activities as well as integrating HIV more explicitly into existing sessions. To reduce the overall intervention length, we merged activities from the two manuals (Stepping Stones and Creating Futures) and reduced some sessions.

To address the challenge of weak and unresponsive health systems for young men, the intervention was implemented within the framework of the ongoing Thetha Nami intervention. As noted above Thetha Nami provided access to youth friendly mobile clinics, and fixed clinics, using a QR code (or their name) for intervention participants. To support linkage to clinics an activity in the intervention included discussion of these clinics, including showing an example of a mobile clinics and what services were on offer. In addition, all participants were provided with an information sheet, which gave details of the services and how to access them, including a number to message to get a call-back.

### Step 2: Pre-test of the intervention

The intervention was then implemented in the short-delivery format, over a two-week period (see Fig 1). The intervention was delivered in AHRI’s main complex in a room with audio privacy by a Project Empower facilitator and two Peer Navigators in attendance. Based on feedback from the short-delivery pre-test, we modified the intervention for the long-delivery format. This included revision of some sessions, and most notably integrating HIV self-testing as an activity directly into the sessions including distribution of four HIV self-test kits per participant.

Uptake and retention of men in the intervention is described in Table 1. Overall, uptake (those agreeing to attend who actually attended one session) was 58%, and retention was 71% (proportion of those who attended one session, attending 6 or more sessions).

**Table 1:** Stepping Stones/Creating Futures attendance and retention.

Stepping Stones Attendance Cascade	Overall	Recruited via random household selection	Recruited via peer networks
	n(%)	n(%)	n(%)
Identified in HDSS	NA	100(100%)	NA
Contacted	NA	71(71%)	NA
Of, those approached, number contacted & recruited	60(64%)	37(52%)	23(100%)
Of those recruited, number attend 1 session (uptake)	35(58%)	12(32%)	23(100%)
Of those who attended 1 session, no. attend 6/9 sessions or more (69%) (retention)	25(71%)	6(50%)	19(83%)

There was variation in uptake and retention by recruitment method with more challenges seen among those randomly selected to participate compared to those recruited via peer networks. Among the 100 men randomly selected to participate only 71 could be identified by the trackers. Of the 29 we could not contact there were two reported deaths, one relocation within the surveillance area, but outside of the area we were working in, and 11 had relocated outside the surveillance area. The other 15 we could not trace at all. Of the 71 participants trackers made contact with, n = 37(51%) agreed to be involved in the study and provided informed consent. Of those who consented to the study, n = 12 (32%) attended at least one intervention session (an overall rate of 12%). Of the 12 who attended at least one session, n = 6 (50%) attended 6/9 sessions (retention).

In total Peer Navigators directly recruited 23 participants meeting the selection criteria who provided informed consent prior to their first session. It is unclear how many men they approached prior to this and how many agreed to attend but did not come to a session. Of the 23 who provided informed consent all attended one session (as informed consent was done shortly before the first session). Of the 23 who attend one session, n = 19(83%) attended at least 6 sessions of the intervention (retention).

There was some suggestion that uptake and retention in the intervention was better among those recruited via peers, rather than through random slection. Specifically, uptake was significantly better in the peer-recruitment (p<0.001, Fishers Exact Test), compared to random recruitment. Similarly, retention was marginally better in the peer recruited group rather than those randomly selected (Table 2). There was no significant difference between retention in the different delivery lengths, although there was a twenty percentage point difference.

**Table 2:** Retention of participants in the intervention by intervention delivery and recruitment.

	All(n = 35)	Short-delivery format (n = 18)	Long-delivery format (n = 17)	p-value^a^	Random selection recruitment (n = 12)	Peer network recruitment (n = 23)	p-value^b^^a^
Retention	n(%)	n(%)	n(%)		n(%)	n(%)	
**0–5 sessions attended**	10 (29%)	3 (17%)	7 (41%)	0.146	6 (50%)	4 (17%)	0.059
**6 or more sessions attended**	25 (71%)	15 (83%)	10 (59%)		6 (50%)	19 (83%)	

^a^ Calculated using Fishers Exact Test

#### Attendance at clinics

Overall, two (6%) of participants who attended at least one session of the intervention went to a mobile clinic within two months of receiving the intervention. In addition, two men tested for HIV and then underwent VMMC at a Department of Health clinic. Finally, in the long-delivery intervention, 56 HIV self-test kits were distributed, to 14 men who attended the session. No additional HIV-tests were recorded within the DoH clinics by participants in the follow-up period.

### Qualitative results

Overall men involved in in-depth interviews had a mean age of 27.2 years, and mean number of sessions attended was six. In total six of the 12 reported HIV-testing after the intervention, though many of these were HIV self-tests (see Table 3).

**Table 3:** In-depth interviewees basic information.

Participant code	Age	Intervention modality	Number of sessions attended (of 9)	HIV-test	Additional information
A	29	short	9	Yes	
B	34	short	5	No	
C	27	short	5	Yes	Then underwent voluntary medical male circumcision
D	32	short	8	No	
E	25	short	9	Yes	Then underwent voluntary medical male circumcision
F	35	short	8	No	
G	23	short	7	No	
H	20	long	8	Yes	Self-test at home
I	21	long	8	Yes	At a clinic
J	22	long	3	No	
K	35	long	5	Yes	Subsequently start ART
L	27	long	6	Yes	Self-test, alongside his girlfriend

#### Experience of the intervention

Men generally found the approach of SSCF acceptable. They described the intervention’s focus on discussion and open debate as something that was different to what they had experienced in previous health interventions:

One of the things that I loved the most about the Stepping Stones workshop is that when I heard that there was a workshop, I already created a picture about the workshop, I honestly thought we were going to have older facilitators. I also had doubts about the programme. When I stepped inside the premises where the workshop took place, I saw my peers, at the same time they are so open minded about everything, we were all entitled to our own opinions without being criticized or looked down on

(Participant 1, short-intervention, FGD)

The men interviewed had primarily been ‘good’ attenders, and they all described how they had enjoyed the sessions and prioritized attendance:

So attending Stepping Stones meant that the time that I normally spent doing other things, I had to sacrifice it for Stepping Stones. However, I do not regret the time I spent attending Stepping Stones because what I have gained, was good for my knowledge.

(Participant F, in-depth interview)

Despite men liking the intervention some did struggle to attend all sessions as they had competing work demands: “*No I did not attend them all*, *I got interrupted…It was just that there was a person that asked me to step in for them at work*” (Participant L, in-depth interview). Another participant described how because he lived at home he sometimes had to do household tasks and could not attend:

I think at times it depends where you live. Some of us still live at home under our parent’s roof. At times it happened we wanted to attend the sessions of the workshop but because we do not live alone, you would find our parents sent us somewhere and it clashed with the workshop.

(Participant 2, long-delivery, FGD).

#### Recognition of importance of health for masculinity and future lives

It was hypothesized that the adapted SSCF intervention would support men to address HIV through getting them to recognize the importance of health for their masculinity and how health would enable them to achieve their future aspirations. The adapted intervention specifically included a focus on young men’s health as an asset men needed: “*So*, *what I learnt was to always look after yourself in order for your future”* (Participant 6, long-format, FGD). Another participant also connected the importance of good health to their future planning:

We spoke about goals, grades, cars, house, wife, money, life, and work. Some stated that if they were to have a car their lives would be good enough. Some stated that if they were to have money, their lives would be good. We continued to have conflicting opinions, we then concluded that in order for you to have a car, you need to be healthy. You can’t get a car when you are lying in bed sick. You can’t get a house or wife, or court a wife or girlfriend when you are sick.

(Participant K, in-depth interview)

#### Knowledge of new HIV-technologies

Many participants had a good understanding of ‘traditional’ HIV prevention strategies, such as condoms and VMMC, and the role of ART in improving life expectancy. However, there was less knowledge about new biomedical technologies, and many men spoke about how they learnt about PrEP and PEP for the first time during SSCF:

Yes, my brother the workshop was able to cover all my needs and concerns as a young man because I could not differentiate between the PreP and PEP pill. I had no idea how they work until they explained it to me thoroughly. Attending this workshop has helped me gain knowledge.

(Participant 1, long-format, FGD)

A second participant also discussed how through participating in SSCF he saw the importance of PrEP in HIV prevention: “*I saw the PrEP pill as the most important topic*, *because it helps you not to contract HIV regardless of whether you have slept with a person is who is infected with HIV/AIDS*”. (Participant 5, short-delivery, FGD)

While most of the discussion was about new biomedical HIV-prevention tools and broadly about the benefit of ART for individual health, a few of the participants discussed how they had found out that ART would reduce HIV-transmission to sexual partners:

Participant: So, what I thought about them was that it is important to know your [HIV] status so you don’t end up getting sick and infecting the person close to you, because you have HIV. It is important that you go and get tested so you can take the treatment so that your viral load can be low.

(Participant L, in-depth interview)

#### HIV as an issue which does not impact on masculinity and can be managed/normalization of HIV

Previous research has highlighted how HIV remains seen as exceptional and impacting on men’s sense of their masculinity [9]. The data after the intervention suggested that there had been a shift in how men thought about HIV towards a normalization of it, as something that could be lived with. As one participant commented, reflecting on what he had learnt from SSCF: *“It was my brother the fact that you can live with this disease [HIV]”* (Participant C, in-depth interview).

Normalisation of HIV in men’s lives was seen in how they described the intervention, as not specifically being only about HIV, but rather all the challenges that men faced:

What was important was there are many things that we learnt. Firstly, I learnt a lot on how to conduct yourself as a guy or when you are in a relationship with a woman. How you manage your anger should you be upset as a guy, as well as where that anger can lead you to. Where a moment of anger can lead you to. Also, the topic of HIV was discussed, even though it is a topic that is always discussed but it helped that when we come here we are able to ask questions directly and freely

(Participant 6, long-delivery, FGD)

The approach of SSCF of integrating HIV into a wider framework of young men’s lives, and this normalizing HIV, was a common theme in interviews:

Interviewer: OK, what would you say were things that were discussed at Stepping Stones that were relevant to your life?

Participant: A lot, it’s things about the life we are living as people in this community, and they might relate to other communities. Yes, it’s things that we go through every day because things like HIV & AIDS, gender-based violence, debts. These are things that define our daily lives.

(Participant C, in-depth interview)

#### Ability to positively manage physical, sexual and mental health

Young men often struggle to prioritise and manage their health proactively. Through SSCF it was anticipated that they would start to see how good health was not only important for their life-goals, but something they needed to actively work towards. There was some limited evidence that this occurred, and men were more proactive in caring for themselves. One way this was seen is how it enabled men to talk about their lives outside the intervention: “*I am now able to open up to other people and receive the services or help I need*. *Although I haven’t received all the help that I need but speaking with other people about my problems relieves me*.” (Participant 4, Rapid Discussion, Short-delivery)

There was also a sense from some participants that it remained important for young men to keep healthy, although this often focused more specifically on HIV, rather than a general sense of maintaining health: “*In my opinion*, *there was nothing difficult because*, *let me say when a person is unwell*, *it helps to know*. *It is not embarrassing because it’s a person’s life*, *and they must know how they take care of themselves*.” (Participant A, in-depth interview).

#### HIV-testing and linkage to care

The ToC suggested through provision of knowledge about new HIV-prevention technologies, men’s recognition of the importance of health for masculinity and their future, the normalization of HIV, as well as a greater ability of men to take charge of their health, HIV-testing would improve and subsequently seek biomedical HIV prevention or treatment. Several participants clearly described how participation in SSCF had supported them go for HIV testing and then seek biomedical interventions to maintain their health. One participant described going to get tested, and then circumcised:

Participant: Yes, HIV test, and the STI one…and I even mentioned that I last tested in 2010.

Interviewer: That’s a very long time ago.

Participant: I went for circumcision [after the intervention], that’s how long ago I last tested…so coming here encouraged me to go get tested. I said to myself if the results are not favorable, then we’ll cross that bridge when we get there. I said let me go get tested and see what happens and then I went and got tested.

(Participant C, in-depth interview)

Another participant similarly described getting an HIV-test following the intervention:

Participant: Yes, I have gone for testing, I was already going for testing, but because it is difficult, you end up not knowing

Interviewer: What made you get tested after the session?

Participant: Uhm the reason was, it is important to know your status

(Participant A, in-depth interview)

In the long-delivery intervention (the second pre-test) HIV self-testing kits were actively distributed to men, whereas in the short-delivery model it was just discussed. Many men in the long-delivery intervention described using the HIV self-testing kits. However, few if any, with an HIV-negative test sought confirmatory testing or accessed additional HIV-prevention technologies. One participant described how he and his girlfriend talked about testing for HIV after the intervention, and that this part of a bigger discussion about their lives and relationship, and subsequently tested using HIV self-tests. However, after testing HIV-negative, they did not then go to a clinic for a confirmatory test or seek other HIV prevention options:

Participant: We tested with that thing [HIV self-test]. We first talked about it.

Interviewer: Okay, have you ever tested before that?

Participant: No, we had not tested.

Interviewer: Oh okay, how long have you been together?

Participant: I think its only been a year.

Interviewer: You went to Stepping Stones, and then you talked about the importance of getting tested and she then agreed with you when you discussed that it is important?

Participant: Yes, she agreed because I explained it to her. The things we learnt about were not only about HIV.

Interviewer: Have you been to the clinic to get tested?

Participant: No, we have not been to the clinic

Interviewer: Oh, how did you get tested?

Participant: Oh, we tested using what was given to us by AHRI.

(Participant L, in-depth interview)

This lack of access to confirmatory HIV testing and access to biomedical HIV prevention technologies was likely driven by several factors. First, some men saw clinics as curative spaces, rather than a tool to promote health, where they would only go if they felt ‘properly’ sick. As one participant said: “*No*, *I haven’t been [to the clinic] because after the programme I haven’t felt sick”* (Participant F, in-depth interview).

Second, almost all men described their great reluctance to go to clinics because of long queues and poor treatment by clinic staff:

Participant: I think the issue is the line, this thing about spending a lot of time at the clinic. You find it is very busy.

Interviewer: Hmmm

Participant: Yes, and sometimes the Doctors there are rude. I tell you. The nurses are rude, yes, they are rude to you.

(Participant G, in-depth interview)

However, in the context of this project men were able to access youth friendly mobile clinics that were part of the wider Theta Nami intervention the Peer Navigators worked on. Mobile clinics were staffed by well trained nurses, with additional training on working with young people, and they had short queues, if any:

They told us about the mobile clinics, and they gave us a leaflet with contact numbers that we can use at anytime. They told us if we want to test, we can go there, usually if you go there it’s just you and no one knows you at mobile clinic.

(Participant C, in-depth interview)

Indeed, one participant who went to the mobile clinic, described a very positive experience, even after testing HIV positive:

Participant: I have not had a bad experience. But what I felt was that when you get there you feel scared, after that the fear goes away. They tested me and told me my results. They then left me there for a while. I then accepted my results because it is important to do so. They also told me that I need to accept that I would be living under these conditions.

Interviewer: Mm-hmm.

Participant: So, and then they… how could I say this? I felt good, they do not mistreat you. Some people may treat you badly however the people there welcomed me. I was happy and felt welcomed. I found my class there.

(Participant K, in-depth interview).

#### On-going trauma of HIV and fear

One issue that SSCF did not adequately tackle was the underlying fear and trauma related to HIV/AIDS many men described. Until the advent and widespread delivery of ART in South Africa, millions of South Africans died of AIDS-related diseases, with KwaZulu-Natal being the epicentre of this. As such many of the young men would have seen the generation before them, their fathers and mothers, dying of AIDS and thus connected HIV and AIDS to death. This left an ongoing fear of HIV in many of the participants, often described in terms of HIV-related stigma. One participant described how the fear and stigma of HIV stopped him going to the clinic:

So we are all sick and at the end of the day I would die because of fear and they live because of their bravery. So, they saw me at the clinic. I stopped whatever I was doing then I went home, while they continued with what they were doing and got their medication.

(Participant F, in-depth interview).

Another participant talked extensively about how he had been scared of testing for HIV, and how SSCF had supported him in overcoming his fear:

Interviewer: You don’t talk?

Participant: It was something I was terrified of

Interviewer: Yes

Participant: Even now, I am still scared of it [Laughs]. Growing up

Interviewer: Hmmm

Participant: It was something I was scared of. I never wanted it near me

Interviewer: Yes

Participant: That is why even if I have just tested, I am happy my brother

(Participant C, in-depth interview).

### Step 3: Finalized ToC and manual

Based on the data collected and analyzed and discussion with the team delivering the intervention we undertook a final revision of the ToC and then the SSCF manual. Into the ToC we integrated the trauma of HIV, and its impact it had on young men’s understanding of HIV. We then revised the manual (see Table 4) integrating a more explicit focus on the trauma of HIV and ensuring HIV self-testing and the provision of HIV self-tests was integral to the process. We included an ongoing WhatsApp discussion group. The finalized manual is 9 sessions long and approximately 48 hours of contact time.

**Table 4:** Manualised Stepping Stones and Creating Futures intervention following pre-tests.

Session	Topic
1: Let’s Communicate	Establishing ground rules, building group relationships, goal setting
2. Situating Self	Establishing goals for self, and
3. How We Act	Gender relationships, body mapping
4. Young men and HIV	Reflect on trauma of loss, new technologies and HIV self-testing (including provision)
5. Education and learning	Support thinking about how to positions ones learning
6. Our social assets: intimate relationships	Relationships, sexuality, power in relationships
7. Social Resources: Our relationships with others	Violence in relationships
8. Earning Money: Jobs and income generating activities	Seeking work and generating ideas
9. Saving, and coping with shocks	Managing money and building assets for the future
10. Reflecting on learning and looking ahead	Summarizing the process, reinforcing goals and next steps

## Discussion

In this discussion we reflect on the extent to which the adapted SSCF met the MRC Framework criteria for a future evaluation, drawing on the data in the co-adapation process and the mixed-methods pre-test of the long- and short-delivery versions of the intervention and recruitment methods.

The pre-test of the modified SSCF intervention showed that the intervention was acceptable for men in both qualitative and quantitative terms. In terms of uptake, two-thirds of men who were offered the intervention attended at least one session. There were, however, differences between those who were recruited via random selection and those via peer networks. Only a third recruited by random selection who agreed to participate, attended any session, while all those recruited via peer networks attended at least one session. In the original SSCF trial, where recruitment was a mixture of community mobilization and peer networks two-thirds (69%, 227/328) who provided informed consent attended at least one session [27]. Ongoing research by AHRI also suggests that peer recruitment may lead to better targeting of people requiring HIV-services; specifically, among 16–30 year old, approximately one-third of those recruited by Peer Navigators were eligible for PrEP, as compared to one in ten using random household selection. Peer recruitment has been widely used among gay men, and sex workers with great effect, and peer support can lead to greater behaviour change [38, 39].

Qualitative data also supported the view that the intervention was acceptable to young men and this was linked to the approach of SSCF. SSCF addressed HIV in a holistic way, and included discussion of young men’s lives, livelihoods, and relationships, as well as HIV. Prior research has suggested the benefit of integrating HIV-programming into wider sexual and reproductive health programming [32, 40].

The intervention was also acceptable as defined by retention during the intervention: with 71% of men who attended one session attending 6 (2/3rds) or more of the sessions. As with uptake, there was an indication that more participants recruited via peer networks were retained than those randomly selected (83% v 50%, although this was only significant at p<0.1). Again, this suggests that peer network recruitment may be more appropriate approach to intervention delivery. In the original SSCF trial only 36% of men who attended one session, attended two-thirds or more sessions, though the intervention was 21 sessions long, with challenges to attendance including work and mobility [27]. There is, however, no definitive understanding of ‘how much’ of an intervention a person is needed to attend to gain benefit from it, and we used two-thirds as the ‘cut-off’ and different definitions of retention could lead to different results.

A factor that supported intervention retention was that men were able to speak about issues of importance to them, as described in the qualitative data. Many of the men’s previous experiences of health education had been didactic lectures instructing men what to do, rather than allowing discussion about these issues [41]. Young men appreciated that SSCF was delivered by someone young, who was like them, and they were actively engaged in debates about things relevant to their lives.

The study showed that it was possible to capture an outcome of attendance at a clinic for an HIV test within the study site, however other important outcomes including HIV self-testing, accessing VMMC and increases in knowledge related to biomedical HIV prevention and treatment were not adequately captured in the outcome focused narrowly on linkage to clinics. One challenge was that while many participants qualitatively reported testing for HIV using self-test kits, there remained a reluctance to access clinics for confirmatory testing amongst those who were negative. This was partly linked to assumptions about clinics being curative, rather than preventative, spaces. As such, strengthening young men’s recognition of health clinics providing prevention (and not only cure) would be an important strengthening of this approach. It may also have been ongoing fear of clinics or continued masculine assumptions of only seeking treatment when sick [9]. An important question for future evaluation of the adapted intervention would be how to capture HIV self-tests and whether linkage to care would be improved because of the intervention in the long-run, as men started to think differently about HIV, masculinity and self-care. Capturing these wider changes over longer periods remains a critical thing to assess as an outcome of an intervention.

There was also broad confirmation of the ToC and mechanisms of change around HIV testing, prevention and treatment, although there remained key gaps that were subsequently addressed in revising the SSCF manual. Often men see HIV as a threat to their identity [9, 42] and there was some evidence that participation in SSCF supported men to see HIV as one of many challenges that they faced in life, rather than ‘the challenge’. Furthermore men seemed to locate HIV as something that needed to be addressed to achieve their other aims. This shift away from ‘AIDS-exceptionalism’ may be important in reducing HIV-related stigma and shifting towards a normalization of HIV in everyday life.

There remained challenges with the underlying ToC, with ongoing community related HIV-stigma and the residual fear of HIV from the high levels of AIDS related deaths in their community in the preceding generations, which may inhibit addressing HIV in their lives [43, 44]. While SSCF did touch on the trauma of AIDS, a greater focus HIV related trauma is warranted and has now been integrated into the ToC and manual (Fig 2, Table 4). Another challenge, particularly because of the shorter intervention period compared to the original SSCF intervention, was the limited time for reflection between sessions and over time, which is the foundational approach of adult educational theories of behaviour change [18].

There was also some evidence that SSCF supported increasing men’s knowledge about HIV-prevention technologies, specifically PrEP and PEP. PrEP was increasingly available through the public sector in South Africa at the time of the intervention, though overall knowledge about PrEP, even in high-risk populations, remained low [2] and men typically did not seek out PreP for themselves. The majority of studies on PrEP have been among women, and gay men, where there is exceedingly high HIV-incidence, though studies in heterosexual women have shown male control may limit PrEP usage [45]. It may be that men, who because of social and economic inequalities have greater autonomy and power, are better placed to take PrEP to reduce HIV-acquisition risk.

### Limitations

This study had a series of limitations. Primarily the pre-test was very small, comprising very few participants and had no control condition to compare to. With recruitment of participants via Peer Navigators, Peer Navigators did not collect data on the number they approached but who refused to participate or agreed to attend but did not attend. As such, we have no data on refusal from peer recruitment to compare with random selection. The qualitative study focused only on men who had attended sessions and we did not seek to understand why men may not have attended, and what could be done to address this. The qualitative study sought to focus on understanding the intervention and what could be done to strengthen the intervention as a process, rather than recruitment approaches. Another significant limitation was the impact of COVID-19 on the process. This limited our ability to have Peer Navigators deliver the intervention, as well as limited some of the co-adaptation time. We do not have any data on other outcomes that SSCF has impacted on, such as IPV perpetration, alcohol use and livelihoods, and it is unclear if the intervention impacted these, and whether similar results would be seen by different delivery mechanism.

## Conclusion

The modified SSCF intervention focused on addressing challenges related to men’s engagement in the HIV prevention and treatment cascade through addressing gender inequitable masculinities and health systems barriers, was promising, with qualitative and quantitative data broadly supporting that the intervention was acceptable and the qualitative data supported the broad mechanism of change and the underlying ToC. Revisions were made to the ToC and manual based on addressing key gaps, with a focus on HIV related trauma and inclusion of HIV self-testing. There remained challenges related to the primary outcome measurement of linkage to clinics, especially for those testing HIV-negative use self-tests. Wider outcomes that recognize knowledge about new biomedical technologies, knowledge of HIV status, and the autonomy that HIV self-testing provides for young men and their sexual partners are also important. More widely previous research on behaviour change interventions addressing masculinity have tended to see change being a gradual process occurring over a few years, rather than an immediate change in behaviour. Evaluating whether the modified SSCF intervention supports men to change their behaviour around HIV-prevention and -treatment over multiple years is a critical outstanding question.

## References

[1] UNAIDS. Blind Spot: Reaching out to men and boys. Geneva: UNAIDS, 2017.

[2] HSRC. South African National HIV Prevalence, Incidence, Behaviour and Communication Survey, 2017. Pretoria, South Africa: HSRC, 2018.

[3] Baisley K, Chimbindi N, Mthiyane N, Floyd S, McGrath N, Pillay D, et al. High HIV incidence and low uptake of HIV prevention services: The context of risk for young male adults prior to DREAMS in rural KwaZulu-Natal, South Africa. Plos One. 2018;13(12):e0208689. doi: 10.1371/journal.pone.0208689 30586376 PMC6306176

[4] Dovel K, Yeatman S, Watkins S, Poulin M. Men’s heightened risk of AIDS-related death: the legacy of gendered HIV testing and treatment strategies. AIDS (London, England). 2015;29(10):1123. doi: 10.1097/QAD.0000000000000655 26035315 PMC4454403

[5] Koo K, Makin JD, Forsyth BW. Barriers to male-partner participation in programs to prevent mother-to-child HIV transmission in South Africa. AIDS Education and Prevention. 2013;25(1):14–24. doi: 10.1521/aeap.2013.25.1.14 23387948 PMC5575863

[6] WHO. Improving men’s uptake of HIV testing and linkage to services: a policy brief. Geneva: WHO, 2021.

[7] Treves-Kagan S, Steward WT, Ntswane L, Haller R, Gilvydis JM, Gulati H, et al. Why increasing availability of ART is not enough: a rapid, community-based study on how HIV-related stigma impacts engagement to care in rural South Africa. BMC Public Health. 2015;16(1):1–13. 10.1186/s12889-016-2753-2 PMC4730651 26823077

[8] Morrell R, Jewkes R, Lindegger G. Hegemonic masculinity/masculinities in South Africa: Culture, power, and gender politics. Men and Masculinities. 2012;15(1):11–30.

[9] Fleming PJ, Colvin C, Peacock D, Dworkin SL. What role can gender-transformative programming for men play in increasing men’s HIV testing and engagement in HIV care and treatment in South Africa? Culture, Health & Sexuality. 2016;18(11):1251–64. doi: 10.1080/13691058.2016.1183045 27267890 PMC5030173

[10] Fleming PJ, Gruskin S, Rojo F, Dworkin SL. Men’s violence against women and men are inter-related: Recommendations for simultaneous intervention. Social Science & Medicine. 2015;146:249–56. doi: 10.1016/j.socscimed.2015.10.021 26482359 PMC4643362

[11] Connell R. Masculinities. Second ed. Cambridge: Polity; 2005.

[12] Fleming PJ, Dworkin SL. The importance of masculinity and gender norms for understanding institutional responses to HIV testing and treatment strategies. AIDS (London, England). 2016;30(1):157. doi: 10.1097/QAD.0000000000000899 26731760 PMC4703363

[13] Adeagbo O, Herbst C, Blandford A, McKendry R, Estcourt C, Seeley J, et al. Exploring people’s candidacy for mobile health–supported HIV testing and care services in rural KwaZulu-Natal, South Africa: Qualitative study. Journal of Medical Internet Research. 2019;21(11):e15681. doi: 10.2196/15681 31738174 PMC6887816

[14] Siu G, Wight D, Seeley J. Masculinity, social context and HIV testing: an ethnographic study of men in Busia district, rural eastern Uganda. BMC Public Health. 2014;14(1):33. doi: 10.1186/1471-2458-14-33 24417763 PMC3893584

[15] Shai N, Sikweyiya Y, van der Heijden I, Abrahams N, Jewkes R. " I was in the darkness but the group brought me light": Development, relevance and feasibility of the Sondela HIV adjustment and coping intervention. Plos One. 2017;12(6):e0178135. doi: 10.1371/journal.pone.0178135 28570597 PMC5453489

[16] Jewkes R, Nduna M, Levin J, Jama N, Dunkle K, Puren A, et al. Impact of Stepping Stones on incidence of HIV and HSV-2 and sexual behaviour in rural South Africa: cluster randomised controlled trial. Brit Med J. 2008;337(7666):-. ISI:000258669200026. doi: 10.1136/bmj.a506 18687720 PMC2505093

[17] Hatcher AM, Colvin CJ, Ndlovu N, Dworkin SL. Intimate partner violence among rural South African men: alcohol use, sexual decision-making, and partner communication. Culture, Health & Sexuality. 2014:1–17. doi: 10.1080/13691058.2014.924558 24939358 PMC4490163

[18] Freire P. Pedagogy of the Oppressed. New York: Continuum; 1973.

[19] Colvin CJ. Strategies for engaging men in HIV services. The Lancet HIV. 2019;6(3):e191–e200. doi: 10.1016/S2352-3018(19)30032-3 30777726

[20] Welbourn A. Stepping Stones: A training package in HIV/AIDS, communication and relationship skills. London: Strategies for Hope, 1995.

[21] Saul J, Bachman G, Allen S, Toiv NF, Cooney C. The DREAMS core package of interventions: A comprehensive approach to preventing HIV among adolescent girls and young women. Plos One. 2018;13(12):e0208167. doi: 10.1371/journal.pone.0208167 30532210 PMC6285267

[22] Skevington S, Sovetkina E, Gillison F. A Systematic Review to Quantitatively Evaluate ‘Stepping Stones’: A Participatory Community-based HIV/AIDS Prevention Intervention. AIDS and Behaviour. 2013;17:1025–39. 10.1007/s10461-012-0327-6 23128978

[23] Gibbs A, Washington L, Abdelatif N, Chirwa E, Willan S, Shai N, et al. Stepping Stones and Creating Futures Intervention to Prevent Intimate Partner Violence Among Young People: Cluster Randomized Controlled Trial. Journal of Adolescent Health. 2020;66:323–35. doi: 10.1016/j.jadohealth.2019.10.004 31784410

[24] Gibbs A, Mkhwanazi S, Sikweyiya Y. Stepping Stones and Creating Futures: a group-based approach to addressing violence against women through working with men. Journal of Clinical Psychology. 2022;78(1):26–37. doi: 10.1002/jclp.23293 34914094 PMC9299760

[25] Gibbs A, Jewkes R, Sikweyiya Y, Willan S. Reconstructing Masculinity? A qualitative evaluation of the Stepping Stones and Creating Futures intervention in urban informal settlements in South Africa Culture, Health & Sexuality. 2015;17(2):208–22. 10.1080/13691058.2014.966150 25335905

[26] Jewkes R, Willan S, Heise L, Washington L, Shai N, Kerr-Wilson A, et al. Effective design and implementation elements in interventions to prevent violence against women and girls. Pretoria: South African Medical Research Council, 2020. 10.3390/ijerph182212129 PMC8621962 34831885

[27] Gibbs A, Dunkle K, Washington L, Sikweyiya Y, Willan S, Shai N, et al. Factors associated with young people’s attendance at an IPV prevention intervention in informal settlements in South Africa: A prospective analysis. Global Public Health. 2020;15(2):161–72. doi: 10.1080/17441692.2019.1662469 31510867

[28] Craig P, Dieppe P, Macintyre S, Michie S, Nazareth I, Petticrew M. Developing and evaluating complex interventions: the new Medical Research Council guidance. BMJ. 2008;337:a1655. doi: 10.1136/bmj.a1655 18824488 PMC2769032

[29] Moore GF, Audrey S, Barker M, Bond L, Bonell C, Hardeman W, et al. Process evaluation of complex interventions: Medical Research Council guidance. bmj. 2015;350:h1258. doi: 10.1136/bmj.h1258 25791983 PMC4366184

[30] Gareta D, Baisley K, Mngomezulu T, Smit T, Khoza T, Nxumalo S, et al. Cohort profile update: Africa Centre Demographic Information System (ACDIS) and population-based HIV survey. International Journal of Epidemiology. 2021;50(1):33. doi: 10.1093/ije/dyaa264 33437994 PMC7938501

[31] Wong EB, Olivier S, Gunda R, Koole O, Surujdeen A, Gareta D, et al. Convergence of infectious and non-communicable disease epidemics in rural South Africa: a cross-sectional, population-based multimorbidity study. The Lancet Global Health. 2021;9(7):e967–e76. doi: 10.1016/S2214-109X(21)00176-5 34143995 PMC8220132

[32] Shahmanesh M, Okesola N, Chimbindi N, Zuma T, Mdluli S, Mthiyane N, et al. Thetha Nami: participatory development of a peer-navigator intervention to deliver biosocial HIV prevention for adolescents and youth in rural South Africa. BMC Public Health. 2021;21(1):1–13. 34256725 10.1186/s12889-021-11399-z PMC8278686

[33] Attride-Stirling J. Thematic networks—an analytical tool for qualitative research. Qualitative Research. 2001;1(3):385–405.

[34] Gibbs A, Jewkes R, Sikweyiya Y. “I tried to resist and avoid bad friends”: The role of social contexts in shaping the transformation of masculinities in a gender-transformative and livelihood strengthening intervention in South Africa. Men and Masculinities. 2019;21(4):501–20. doi: 10.1177/1097184X17696173

[35] Gibbs A, Jewkes R, Willan S, Washington L. Associations between poverty, mental health and substance use, gender power, and intimate partner violence amongst young (18–30) women and men in urban informal settlements in South Africa: A cross-sectional study and structural equation model. Plos One. 2018;13(10):e0204956. doi: 10.1371/journal.pone.0204956 30281677 PMC6169941

[36] Kerr-Wilson A, Gibbs A, McAslan Fraser E, Ramsoomar L, Parke A, Khuwaja H, et al. What works to prevent violence against women and girls? A rigorous global evidence review of interventions to prevent violence against women and girls. Pretoria, South Africa: What Works to Prevent Violence Against Women and Girls, 2020.

[37] Shahmanesh M, Mthiyane TN, Herbsst C, Neuman M, Adeagbo O, Mee P, et al. Effect of peer-distributed HIV self-test kits on demand for biomedical HIV prevention in rural KwaZulu-Natal, South Africa: a three-armed cluster-randomised trial comparing social networks versus direct delivery. BMJ Global Health. 2021;6(Suppl 4):e004574. doi: 10.1136/bmjgh-2020-004574 34315730 PMC8317107

[38] Sokol R, Fisher E. Peer support for the hardly reached: a systematic review. American Journal of Public Health. 2016;106(7):e1–e8. doi: 10.2105/AJPH.2016.303180 27196645 PMC4984766

[39] Genberg BL, Shangani S, Sabatino K, Rachlis B, Wachira J, Braitstein P, et al. Improving engagement in the HIV care cascade: a systematic review of interventions involving people living with HIV/AIDS as peers. AIDS and Behavior. 2016;20(10):2452–63. doi: 10.1007/s10461-016-1307-z 26837630 PMC4970970

[40] Bulstra CA, Hontelez JA, Otto M, Stepanova A, Lamontagne E, Yakusik A, et al. Integrating HIV services and other health services: A systematic review and meta-analysis. PLoS medicine. 2021;18(11):e1003836. 34752477 10.1371/journal.pmed.1003836 PMC8577772

[41] Campbell C, MacPhail C. Peer education, gender and the development of critical consciousness: Participatory HIV prevention by South African youth. Soc Sci Med. 2002;55(2):331–45. doi: 10.1016/s0277-9536(01)00289-1 12144146

[42] Sileo KM, Reed E, Kizito W, Wagman JA, Stockman JK, Wanyenze RK, et al. Masculinity and engagement in HIV care among male fisherfolk on HIV treatment in Uganda. Culture, Health & Sexuality. 2019;21(7):774–88. doi: 10.1080/13691058.2018.1516299 30422078 PMC6513725

[43] Moyer E, Hardon A. A disease unlike any other? Why HIV remains exceptional in the age of treatment. Medical Anthropology. 2014;33(4):263–9. doi: 10.1080/01459740.2014.890618 24661122

[44] Cluver L, Gardner F, Operario D. Psychological distress amongst AIDS-orphaned children in urban South Africa. Journal of Child Psychology and Psychiatry. 2007;48(8):755–63. doi: 10.1111/j.1469-7610.2007.01757.x 17683447

[45] Jani N, Mathur S, Kahabuka C, Makyao N, Pilgrim N. Relationship dynamics and anticipated stigma: Key considerations for PrEP use among Tanzanian adolescent girls and young women and male partners. Plos One. 2021;16(2):e0246717. doi: 10.1371/journal.pone.0246717 33596216 PMC7888654

